# Comment on “Comparative risk assessment of cardiovascular and kidney outcomes in patients with type 2 diabetes using cystatin C and creatinine in DECLARE-TIMI 58”

**DOI:** 10.1016/j.ahjo.2026.100963

**Published:** 2026-10-15

**Authors:** A. Kala, E. Shanma, Moneekha Priyadarshini, D. Anandhi

**Affiliations:** a Obstetrics and Gynaecology, Meenakshi Medical College Hospital & Research Institute, Meenakshi Academy of Higher Education and Research, Enathur, Kanchipuram, Tamil Nadu, 631552, India; b Obstetrics and Gynaecology, Meenakshi Medical College Hospital & Research Institute, Meenakshi Academy of Higher Education and Research Enathur, Kanchipuram, Tamil Nadu, 631552, India; c Department of Biochemistry, Meenakshi Ammal Dental College and Hospital, Meenakshi Academy of Higher Education and Research, Chennai, Tamil Nadu, India

**Keywords:** Type 2 diabetes mellitus, Cystatin C, Serum creatinine, Cardiovascular outcomes, Kidney outcomes, DECLARE-TIMI 58

Dear Editor

Small et al. [1], report that cystatin C (CysC) showed a gradient in cardiovascular and kidney risk among 1899 placebo-treated participants in the DECLARE-TIMI 58 trial while creatinine-based measures did not. They also found that dapagliflozin had effects across different CysC levels. We agree that CysC may carry risk information but we believe the comparison with creatinine was unfairly biased from the start and the paper asks more of the creatinine data than the trial design can reasonably support.

The trial enrolled patients only if their creatinine clearance was least 60 mL/min. That selection criterion was based on creatinine not on cystatin C. As a result the creatinine-based eGFR distribution was sharply truncated. 10.1% Of the analysis cohort had an eGFR below 60 mL/min using creatinine while CysC retained its full range—from 0.2 to 3.7 mg/L. A marker that has been filtered by the study's inclusion rules will naturally show a narrower gradient no matter how well it reflects kidney function [2]. So the fact that creatinine gradients appeared attenuated may simply reflect the design, not a true difference in performance between the two markers.

The paper relies heavily on *P* values to make its point. The trend *P* value for CysC was than .001 while for creatinine it was .34. A significant *P* value for one marker and a nonsignificant one for the other does not prove that the markers differ [3]. For CysC the hazard ratios comparing the top versus bottom quartile were 1.33 (95% CI 0.98 to 1.80) for the heart failure composite and 1.95 (95% CI 0.95 to 3.99) for kidney outcomes. These confidence intervals cross 1.0 meaning the differences are not statistically robust [4]. The c-statistics were quite similar between markers and the category-free net reclassification improvement (NRI) included zero for both the heart failure composite and kidney outcomes. For major adverse cardiovascular events (MACE) did the interval exclude zero (0.11, 95% CI 0.02 to 0.20). To truly compare the markers one would need to include both in a model or test the difference in hazard ratios per standard deviation—this would give a direct answer to the question.

We also note that CysC may be capturing more than kidney function. Across quartiles median BMI increased from 30.5 to 34.2 prior heart failure rose from 10.0% to 22.3% and insulin use went from 38.7% to 57.3%. The discussion acknowledges that adiposity and inflammation affect levels independently of glomerular filtration rate (GFR). The models adjusted for BMI and heart failure history but did not control for any markers. This means part of the signal could reflect underlying cardiometabolic risk rather than a better measurement of kidney function. CysC may still be a risk marker but that does not mean it is necessarily the superior marker for kidney function as the introduction suggests.

Some methodological details are missing. The study is described as a case-cohort design. When using Cox models and Harrell's C in such designs sampling weights are required to account for the sampling process. These weights are not described. The analysis refers to plasma samples. Cysc is reported as a serum measurement. This inconsistency needs clarification. Also the claim that dapagliflozin's effect was consistent across CysC levels is based on interaction *P* values above.05 in the same case-cohort sample with the top and bottom 5% of CysC values excluded from the spline plot. A nonsignificant interaction is not proof of benefit especially, at higher CysC levels, where absolute risk is greatest and the clinical impact could be most important.

Cystatin C may still have value. To really test whether it outperforms creatinine we would need to see it used in a population not selected based on creatinine. Then can we judge whether CysC provides a truer or more sensitive reflection of kidney function and risk.

## CRediT authorship contribution statement

**A. Kala:** Writing – review & editing, Writing – original draft, Visualization. **E. Shanma:** Conceptualization, Data curation, Formal analysis. **Moneekha Priyadarshini:** Writing – review & editing, Writing – original draft, Visualization. **D. Anandhi:** Writing – review & editing, Writing – original draft, Visualization.

## Ethical approval

Not applicable (commentary on previously published research).

## Declaration of Generative AI and AI-assisted technologies in the writing process

Generative AI tools were used solely for language refinement and formatting assistance. All scientific interpretation, critique, and conceptual analysis were independently developed by the authors.

## Funding

None.

## Declaration of competing interest

The authors declare no conflicts of interest.

## Data Availability

No new data were generated or analysed in this work.
